# Resistance exercise effects on hippocampus subfield volumes and biomarkers of neuroplasticity and neuroinflammation in older adults with low and high risk of mild cognitive impairment: a randomized controlled trial

**DOI:** 10.1007/s11357-024-01110-6

**Published:** 2024-03-13

**Authors:** Wouter A. J. Vints, Julija Šeikinaitė, Evrim Gökçe, Simona Kušleikienė, Milda Šarkinaite, Kristina Valatkeviciene, Vida J. Česnaitienė, Jeanine Verbunt, Oron Levin, Nerijus Masiulis

**Affiliations:** 1 Department of Health Promotion and Rehabilitation, Lithuanian Sports University, Kaunas, Lithuania; 2 Department of Rehabilitation Medicine Research School CAPHRI, Maastricht University, Maastricht, The Netherlands; 3 Centre of Expertise in Rehabilitation and Audiology, Adelante Zorggroep, Hoensbroek, The Netherlands; 4 Department of Rehabilitation, Physical and Sports Medicine, Institute of Health Science, Vilnius University, Vilnius, Lithuania; 5 Sports Rehabilitation Laboratory, Ankara City Hospital, 06800 Ankara, Turkey; 6 Department of Radiology, Medical Academy, Lithuanian University of Health Sciences, Kaunas, Lithuania; 7 Motor Control & Neuroplasticity Research Group, Group Biomedical Sciences, Catholic University Leuven, Heverlee, Belgium

**Keywords:** Hippocampus, Cognitive aging, Myokines, Resistance exercise, Neurotrophic factor, Inflammation

## Abstract

Physical exercise is suggested to promote hippocampal neuroplasticity by increasing circulating neurotrophic and anti-inflammatory factors. Our aim was to explore the interplay between the effect of progressive resistance exercise on blood biomarker levels, hippocampal neurometabolite levels and hippocampal volume in older adults with a low compared to a high risk of mild cognitive impairment (MCI). Seventy apparently healthy male/female older adults (aged 60–85 years old) were randomly allocated to a 12 week lower limb progressive resistance or no intervention, stratified for low (< 26/30) or high (≥ 26/30) Montreal Cognitive Assessment (MoCA) score, indicating MCI risk. Outcome measures were blood levels of insulin-like growth factor-1 (IGF-1), interleukin-6 (IL-6) or kynurenine (KYN); hippocampal total and subfield volumes of the cornu ammonis 1 (CA1) and 4 (CA4), subiculum, presubiculum, and dentate gyrus measured with magnetic resonance imaging (MRI); and hippocampus neurometabolites including total N-acetylaspartate (NAA), myo-inositol (mIns), and total creatine (Cr) measured with proton magnetic resonance spectroscopy (^1^H-MRS). We evaluated the intervention effect, cognitive status effect, their interaction and the bivariate relationship between exercise-induced changes between the outcome measures. Higher kynurenine levels (p = 0.015) and lower subiculum volumes (p = 0.043) were found in older adults with high MCI risk compared to older adults with low MCI risk. Exercise-induced CA1 volume changes were negatively correlated with hippocampal tNAA/mIns level changes (r = -0.605, p = 0.006). This study provides valuable insight in the multifactorial processes related to resistance training in older adults with low or high MCI risk.

**Supplementary Information:**

The online version contains supplementary material available at 10.1007/s11357-024-01110-6.

## Introduction

The hippocampus is a brain region with a substantial capacity for structural reorganization, or neuroplasticity. It can rapidly modify existing neural circuits and even create entirely novel neural connections through the process of neurogenesis [1]. Specifically, the dentate gyrus (DG) of the hippocampus is known for its continued ability to generate new neurons throughout life [2]. Importantly, the neurogenetic potential of the hippocampus seems to be highly responsive to external stimuli. For example, hippocampal neurogenesis and neuroplastic processes are facilitated in response to physical activity [3], while they are impaired by stress, alcohol, and sleep deprivation [4, 5]. Furthermore, studies in older adults show significant age-related decreases in hippocampal neuroplasticity and hippocampal volume, associated with age-related cognitive decline [6, 7]. Hippocampal volume loss can precede cognitive impairment by several years [8], and in older adults with mild cognitive impairment (MCI) severe losses in the cornu ammonis subfield 1 (CA1) and subiculum hippocampal subfields predict progression towards Alzheimer’s dementia [9–13].

It has been suggested that the hippocampal neurogenetic and neuroplastic potential is modulated by several neurotrophic and inflammatory markers [14]. In older adults, a state of low-grade inflammation, referred to as ‘inflamm-aging’ [15], is thought to impair hippocampal plasticity [14, 16]. With inflamm-aging, old and damaged cells throughout the body start to release inflammatory cytokines, such as interleukin-6 (IL-6), into the blood stream. The number of these senescent cells gradually increases with aging [17] resulting in an increase in pro-inflammatory factors that can cross the blood–brain barrier. This, in turn, increases the numbers and pro-inflammatory activity of microglia in the hippocampus and other brain regions, leading to impairments in memory and executive control functions [18]. Increased microglial activity and its effect on brain’s structural and neurochemical properties can be estimated in-vivo with various neuroimaging techniques. For example, proton magnetic resonance spectroscopy (^1^H-MRS) studies overall report that older adults, especially cases with MCI who are at high risk of converting into Alzheimer’s disease (AD), have increased levels of the neurometabolites myo-inositol (mIns), a marker for microglial cell density, and later on also decreased levels of N-acetylaspartate (NAA), a marker for neural density [19–21]. Specifically, decreased levels of NAA and/or decreased ratio of NAA to creatine (Cr) have been proposed as markers of brain atrophy, increased levels of myo-inositol (mIns) and/or mIns/Cr are proposed to mark neuroinflammation, whereas decreased ratio of NAA/mIns has been proposed as a combined marker of neurodegeneration and neuroinflammation [22]. Increased brain mIns levels may be found in preclinical Alzheimer’s disease, and even precede detectability of amyloid β in cerebrospinal fluid [23, 24], which had been reported before as the first biomarker to become abnormal in Alzheimer’s disease [25]. Interestingly, we previously showed that circulating levels of the inflammatory marker kynurenine (KYN) were associated with levels of neuroinflammation and neurodegeneration markers measured with ^1^H-MRS in older adults [21]. Specifically, elevated levels of serum KYN were associated with signs of neurodegeneration (i.e., decreased levels of NAA) in hippocampus and medial temporal cortex and signs of neuroinflammation (i.e., increased levels of mIns) in the posterior cingulate cortex and dorsolateral prefrontal cortex. KYN is of particular interest in the context of hippocampal neuroinflammation, since elevated circulatory KYN levels were associated with reduced memory function in older adults [26]. Moreover, the activity of the enzyme responsible for the upregulation of KYN levels is increased by pro-inflammatory cytokines, making KYN a generic marker of pro-inflammatory cytokine activity [27].

The use of physical activity as a nonpharmacological treatment to mitigate pro-inflammatory processes and increase the neuroplastic capacity of the hippocampus present an exciting avenue for healthy aging interventions [4]. As we mentioned before, physical exercise was reported to enhance hippocampal neurogenetic potential [28], but also facilitate other neuroplastic processes such as long-term synaptic potentiation (LTP) [16], increase hippocampal volume, and improve memory function [3]. For example, cytokines and peptides released from muscle tissue during exercise, such as IL-6 which was introduced by Pedersen and colleagues as the first “myokine” [29], are argued to play a role as mediators of exercise-induced beneficial effects on neurogenesis and cognition [16, 30]. Furthermore, it was discovered that exercise increases muscle expression of kynurenine aminotransferase, an enzyme that converts KYN into kynurenic acid. In contrast to KYN, kynurenic acid is no longer capable of crossing the blood–brain barrier and provoking pro-inflammatory processes in the brain [31]. Instead, kynurenic acid has anti-inflammatory and neuroprotective effects [32]. In general, however, a bout of physical exercise transiently increases inflammatory markers, like IL-6, and neurotrophic factors, such as insulin-like growth factor-1 (IGF-1), while chronic exercise interventions result in decreased levels of inflammatory markers and increased levels of neurotrophic factors [16]. This exercise-effect has been shown for various exercise modes and doses. However, some authors suggest that resistance exercise may cause larger changes in neurotrophic and inflammatory blood biomarkers [33–38]. It should be noted, however, that more research has been done with endurance exercise compared to resistance exercise, and the variability in reported findings between studies may as well depend on other study characteristics than exercise mode. For example, IGF-1 was particularly increased by resistance exercise in older adults, but to a lesser extent in younger adults [35, 39, 40]. To our knowledge, the interrelationship between resistance exercise, blood biomarkers, hippocampal neurometabolites, and hippocampal subfield volumes has never been explored in detail, with the hippocampus being considered as primary brain area for neurogenesis but also a brain structure vulnerable to pro-inflammatory conditions leading to cognitive declines [1, 4, 5]. Additionally, the comparison between cognitively healthy older adults with low risk of MCI and older adults with near-normal cognitive performance who are at high risk of MCI would provide valuable novel information for the research field.

## Objectives and hypotheses

The primary objective of this randomized controlled trial was to explore the effect of chronic resistance training on the hippocampus in a population of older adults with normal cognitive function or with probable MCI. Specifically, to examine the changes induced by 12 weeks of resistance exercise on blood biomarker levels, hippocampal neurometabolite levels and hippocampal volume and the interplay between those changes. Resistance exercise effects were compared to time effects seen in a waiting list control condition. The outcome measures encompassed blood circulating markers (IL-6, KYN, and IGF-1) and brain neurometabolic markers (tNAA/tCr, mIns/tCr, and tNAA/mIns) that have been identified as biomarkers related to neuroplasticity and neuroinflammation [14, 16, 22], as well as total and subfield hippocampus volumes. Our second objective was to explore the influence of cognitive status at baseline on these biomarkers and the resistance exercise effect. In consideration of our objectives, we formulated a hypothesis that (1) resistance exercise would induce increases in the neurotrophic blood biomarker IGF-1 and neurometabolites tNAA/tCr and tNAA/mIns in the hippocampus and decreases in the inflammatory blood biomarkers IL-6 and KYN and brain biomarker mIns/tCr in the hippocampus. Furthermore, we hypothesized that (2) resistance exercise would induce an increase in total and subfield hippocampus volumes. Moreover, we hypothesized that (3) the resistance exercise-induced changes in these biomarkers and hippocampus volumes would be interrelated. Finally, we hypothesized that (4) the changes induced by resistance exercise would be larger in older adults with high risk of MCI compared to their low-risk peers. This knowledge may serve to design interventions aimed at preventing the age-related deterioration of brain health.

## Methods

### Participants and setting

Seventy older adults (male/female, 32/38) aged 60 to 85 years participated in a randomized controlled trial. Participants were recruited between July 2020 and July 2021 from volunteers from previous studies, a list of patients provided by general practitioners, and presentations in local community organizations in Kaunas, Lithuania. The exercise-induced changes in muscular strength from a subgroup of these participants and how they relate to neurometabolic changes has been published previously [41]. In addition, the baseline balance, physical fitness, and body composition measurements have previously been evaluated in relation to baseline blood biomarker levels, brain health markers, cognitive function tests and balance control [21, 42–44]. In comparison to the studies published earlier, four participants were deleted. Two of these participants underwent baseline assessments, but they participated in a pilot version of the current intervention. After their participation, the final version of the resistance exercise protocol was decided. Two other participants did not continue after baseline assessments, either because of a claustrophobic attack during magnetic resonance imaging (MRI) or because of a pathological finding on brain MRI scanning.

Exclusion criteria were alcohol or drug abuse, neurologic, oncologic or psychiatric diagnosis, use of psychopharmacological drugs in the last five years, or a history of chemotherapy. Participants were excluded if they had signs or symptoms of cardiovascular, pulmonary or metabolic diseases, with the only exception that some of the participants had hypertension. The mean blood pressure and heart rate of the included participants before intervention were respectively 139/78 (range systolic blood pressure: 108–182; range diastolic blood pressure: 55–119) and 70 bpm (range: 52-96 bpm). These measurements were done in a seated position after 30 min of seated rest. Participants had to be physically healthy and able to perform ten sit-ups. They had to be allowed to undergo MRI according to the checklist provided by the Department of Radiology at the Lithuanian University of Health Science. Finally, we excluded participants that participated in an exercise program on a regular basis in the last six months. Participants were allowed to withdraw from the study at any time. The study methods were approved by the Kaunas Regional Biomedical Research Ethics Committee (No. BE-10–7) and a written informed consent was obtained from all participants prior to their inclusion in the study.

### Study design

This study was a parallel group randomized controlled trial with a 12-week resistance exercise intervention. Randomization was done using a stratified permuted block procedure. The stratification factor was the participants score on the Montreal Cognitive Assessment (MoCA) test, subdividing participants into high and low risk of MCI. MoCA tests were conducted by a qualified mental-health care specialist (co-author SK). While the MoCA test is not a diagnostic tool, a score of 18–25 is considered a reliable and sensitive marker of MCI, whereas a score of 26 to 30 is considered to indicate normal cognitive ability and low risk for MCI [45, 46]. The MoCA test scores from the participants ranged from 19–30. In every block of four participants with low or high risk of MCI, two were randomly allocated to the experimental and two to the control condition. This allocation was done in an Excel spreadsheet, using a random number generator set to indicate either 1 or 2 for exercise or control group respectively. If a block of four participants (N) with the same cognitive status contained two participants of the same group, the third participant was allocated to block N + 1 with this cognitive status. In the last block of eight participants, only high MCI risk participants were included and seven out of eight participants were allocated to the control group. The reason for this decision was to correct for a higher number of drop-outs in the high MCI risk control groups at that time during the project. A participant flow diagram is presented in Appendix B, Supplementary Fig. 1. The resistance training protocol was supervised by qualified fitness coaches, who were not involved in pre-and post-intervention data collection. The investigators involved in data collection were blinded to the group allocation.

### Assessments

**Fig. 1:**
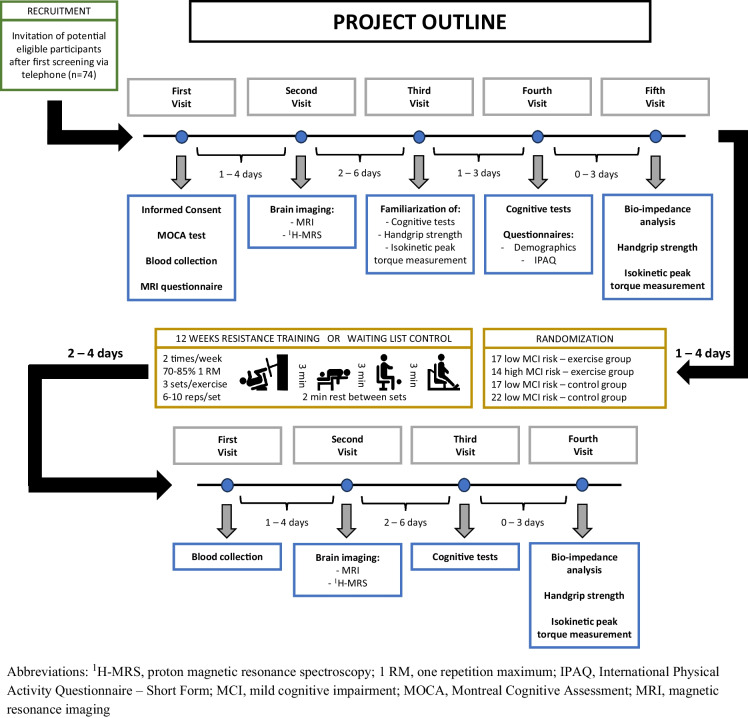
Project outline

An overview of the timing of all assessments and summary of the interventional protocol is presented in Fig. 1.

#### Demographic and clinical characteristics

Participants were asked to complete a demographics questionnaire assessing their age, sex, educational level (categorized as basic education, secondary education or higher education), and smoking status. Self-reported physical activity level was estimated using the International Physical Activity Questionnaire – Short Form (IPAQ-SF). Physical activity level is calculated based on the total kcal burned per week during exercise of light, moderate or vigorous intensity, using the formula: total kcal/week = the sum of days performing light/moderate/vigorous activity × average time/day performing these activities × F, where F equals 3.3 for light intense exercise, 4.0 for moderate intense exercise and 8.0 for vigorous intense exercise. Participants burning less than 600 kcal per week were categorized as sedentary, 600–3000 kcal per week as moderately active and > 3000 kcal per week as highly active [47].

All participants’ body mass index (BMI) and body fat percentage (fat%) were measured on a leg-to-leg bio-impedance analyser (Tanita TBF-300-A). After a 5 min warm-up by pedalling at an intensity of 60–90 Watts on a veloergometer, and 3 min of dynamic activation exercises including lunges, butt kicks, side step lunges, half-squats, and front and side cross swings, isometric knee extension torque on maximum voluntary contraction (MVC, in Newton meters) of the dominant leg was measured with a Biodex System 3 dynamometer (Biodex Medical Systems, NY, USA). The highest value out of two trials was used for analysis. The MVC was only measured in a subgroup of the participants (45 participants pre-intervention and 20 post-intervention; 8 from the control and 12 from the exercise group). This subgroup was analysed in study of Sheoran et al. [41]. Handgrip strength (in kg) was measured using a JAMAR 11940248 adjustable hand grip strength testing system in standing position. The grip size was adjusted so that there was a 90° angle in the second joint of the index finger. The test was preceded by a first try at submaximal effort, followed by 2 tries at maximal effort with 1 min interval between trials and the highest value was used for analysis.

#### Blood serum analysis

All blood samples were drawn between 9 a.m. and 1 p.m. by a qualified medical professional. For participants in the experimental group, the second blood collection took place at least 48 h or more after the last exercise bout. Blood was collected in 5 mL serum separator tubes at the antecubital vein. The samples were gently inverted 8–10 times and stored for 30 min at room temperature to allow clotting. Subsequently, the tubes were centrifugated for 15 min at 4000 g. From these samples, blood serum was aliquoted into 1.5 mL polypropylene tubes and stored until further analysis in a -80 °C refrigerator compartment at the laboratory of the Lithuanian Sports University. After full completion of the study, the samples were analysed with enzyme-linked immunosorbent assays (ELISA) using a spectrophotometer (Spark 10 M, Tecan Group Ltd., Zürich, Switzerland) by an experienced lab technician. The following blood biomarkers were measured using commercially available ELISA kits: IGF-1 (IBL International, GMBH, Germany, MD58011, with a lower limit of detection 0.03 ng/mL), IL-6 (DIAsource ImmunoAssays S.A., Belgium, KAP1216, with a lower limit of detection 2 pg/mL), KYN (MyBiosource, Inc., USA, with lower limit of detection 45.7 ng/mL).

#### Hippocampus volume and neurometabolites

Participants underwent whole brain MRI and ^1^H-MRS scanning in a 3 Tesla Skyra scanner (Siemens Healthineers, Erlangen, Germany) with a 32-channel receiver head coil. Total scanning duration was 90 min per participant. Only hippocampus measurements are presented in this paper.

Pre- and post-intervention hippocampus total and subfield grey matter volumes (GMV) were calculated using 3D magnetization prepared gradient echo (MPRAGE) images from high resolution T1-weighted structural MRI (repetition time (TR) = 2,200 ms, echo time (TE) = 2,48 ms, voxel size 0.9 × 0.9 × 1.0 mm^3^, field of view 230 × 256 mm, number of sagittal slices = 176). A longitudinal analysis pipeline of FreeSurfer v7.1.1 (Harvard, MA, USA, http://surfer.nmr.mgh.harvard.edu/) was used to measure the volumes of the left hippocampal subfields. The following subfields were examined: left whole hippocampus, left CA1 body and head (combined the left CA1), left subiculum body and head (combined the left subiculum), left presubiculum body and head (combined the left presubiculum), the left CA4 body and head (combined the left CA4), and the left granule cell (GC) and molecular layer (ML) of the dentate gyrus (DG) (combined the DG).

Hippocampus ^1^H-MRS spectra were acquired using a Point RESolved Spectroscopy (PRESS) sequence (TR = 2,000 ms, TE = 30 ms, number of averages = 128, spectral bandwidth = 2,000 Hz, and data size = 1,024 points) with chemical shift selective (CHESS) water suppression (bandwidth = 50 Hz) and a voxel size of 26 × 12 × 12 cm^3^. The hippocampal voxel was centered over the whole hippocampus in left hemisphere in the medial part of the temporal lobe anterior to lateral ventricle, corresponding to the left whole hippocampus Freesurfer region. Voxel position and an example of a post-processed spectrum are presented in Appendix A, Supplementary Fig. 2. The acquired spectra were processed with linear combination of model spectra (LCModel, version 6.3.1-R) postprocessing software. Spectra with Cramér-Rao lower bound ≥ 20%, linewidths ≥ 12 Hz and signal-to-noise ratio < 5 were excluded. All ^1^H-MRS spectra were visually checked to ensure the absence of artifacts prior to quantification. In this paper, we reported results from total NAA (tNAA) composed of N-acetyl aspartate and N-acetyl glutamate, mIns, and their ratio to total Cr (tCr) composed of creatine and phosphocreatine. In addition, the ratio of tNAA relative to mIns was calculated. A detailed overview of the ^1^H-MRS methods according to the minimum reporting standards of the MRSinMRS experts’ consensus recommendations can be found in Appendix B [48].

### Intervention: resistance exercise intervention

Participants in the exercise group took part in a 12-week progressive resistance exercise program for the lower limbs, while participants in the control group did not receive any treatment and were asked not to change their physical activity levels until post-intervention outcome measurements. The resistance exercise protocol was in line with latest position statement by the National Strength and Conditioning Association (USA) for resistance training for older adults [49]. Older adults in the exercise group trained two times per week under direct supervision of professional fitness instructors, with maximum two participants per fitness instructor at the same time. The training sessions took place in the gym at the Lithuanian Sports University using resistance training equipment from Technogym (Italy). Warm-up consisted of 5-min cycling on a cycloergometer, at an intensity (in Watts) approximately equal to the participants’ body weight in kilograms, followed by a few dynamic stretching and activation exercises including lunges, butt kicks, side step lunges, half-squats, and front and side cross swings. The main resistance exercise program consisted of three sets of four lower limb exercises, (1) leg press, (2) leg curl, (3) leg extension, and (4) calf raises. The order of these exercises was not controlled, but in general the participants were instructed to start with the leg press as this is a multi-joint exercise and end with a single-joint exercise such as the calf raises. Lower limb strength and power are critical determinants of physical functioning in older adults, and correlate with their overall well-being [50]. Moreover, age-related muscle atrophy was observed to mainly affect the lower limbs, while upper limb muscles remain relatively unaffected [51]. In the first week of training, the older adults were familiarized with the exercise movements and underwent a1-repetition maximum (1-RM) test for all four exercises. The 1-RM assessment was based on a standard protocol recommended by the National Strength and Conditioning Association [52]. It involved a prediction calculation based on the number of repetitions performed at submaximal loads which was performed using the ExRx.net calculator (https://exrx.net/Calculators/OneRepMax) [53]. In the remaining weeks, participants in the exercise group did one warm-up set and three working sets for all four lower limb exercises, with a rest of 2 min between sets and 3 min between exercises. From week 1 till 3, participants did 8–10 repetitions at 70–75% 1-RM, from week 4 till 9 they worked at 6–8 repetitions at 75–80% 1-RM, and from week 10 till 12 they did 6 repetitions at 80–85% 1-RM. The weight was adjusted during the three training blocks according to the participants’ rate of perceived exertion (RPE) on a 10-point Borg scale [54]. The weight was increased when the older adult indicated a score below 7 on 10. The RPE was logged in a notebook by the fitness instructors, along with the number of repetitions and the weight lifted.

### Statistical analysis

IBM SPSS Statistics version 27 (IBM Inc., Chicago, USA) was used to perform all analyses. First, the data was inspected for outliers and normality. Extreme outliers, defined as values lying more than 3 × the interquartile range away from the median were excluded. Normality was defined as a kurtosis and skewness measure between -2 and + 2. Additionally, normality was checked visually using PP-plots and histograms. Data not meeting one of the normality assumptions was log transformed. Homoskedasticity was tested with the Levene’s test.

Group differences in baseline descriptives were analysed using independent t-tests and Chi^2^ tests (or Fisher Exact tests, if the expected count in any of the cells was below 5) for continuous and categorical variable respectively. Our primary research question, the evaluation of the relationship between changes in outcome measurements from baseline to 12 weeks later, was measured using bivariate correlations. R-values based on Spearman’s rho were chosen, given the non-normal distribution of some of the outcome measures. Our secondary objectives, to test the effect of exercise compared to control, the influence of cognitive status and their interaction, were evaluated using two-way ANCOVA with either blood biomarker levels, hippocampal neurometabolites or hippocampal subfield volume results at the 12 week measurement point as the dependent variable. Group (experimental versus control) and cognitive status (low MCI risk versus high MCI risk) were entered as fixed factors, and age and pretest values of the dependent variable as covariates. For analysis with IL-6 or KYN levels as dependent variables, fat% was entered as an additional covariate given that inflammatory markers are known to be moderated by individuals’ fat% [55, 56]. For analysis with hippocampus (subfield) volumes, intracranial volume was entered as an additional covariate in order to adjust the values for head size. The intracranial volumes measured at baseline and after 12 weeks were identical for all participants. We chose for a two-way ANCOVA instead of a three-way repeated measures ANOVA, because it was shown to reduce the population error variance and increase the power and preciseness of the test. The conclusion of these two test is generally the same when the ANCOVA tests takes into account the pretest value of the dependent variable by entering it as a covariate in the model, compared to adding it as a level of the time factor in repeated measures ANOVA. For further reading, see Rausch et al. [57].

## Results

### Participants characteristics

52 participants (74.3%) completed the intervention. Reasons for dropping out were COVID-19 or fear of catching a SARS-COV-2 infection, lack of motivation, and intervention related trauma or fear of injury. The descriptive values of the baseline characteristics for exercise group, control group and total sample are presented in Appendix A, Supplementary Table 1. There was a baseline difference between the two groups for the amount of kilocalories burned with physical activity per week (*p* = 0.024) and educational level (*p* = 0.038). The mean group change from baseline to 12 weeks later in knee extension MVC in the control group (*n* = 8) was -1.7 Nm (*SD* = 8.7 Nm), compared to + 31.7 Nm (SD = 41.3 Nm) in the experimental group (*n* = 12) (*p* = 0.038). There were no significant differences between groups for blood or left hippocampus (subfield) volumes and neurometabolite levels at baseline. The values are presented in Tables 1, 2 and 3. Finally, there were no significant differences between older adults with low MCI risk and high MCI risk for any of the baseline measurements (see Supplementary Table 2).

**Table 1:** Descriptive values of baseline/12 weeks blood biomarker levels and percentage change

		BASELINE	12 WEEKS	Δ (%)
IGF-1 (ng/mL)	EXP (*n* = 24) -hrMCI (*n* = 12) -lrMCI (*n* = 12)	119.0 (57.6) 118.0 (55.2) 120.1 (62.4)	139.2 (85.4) 143.2 (81.2) 135.3 (92.8)	+ 17.0 + 21.4 + 12.7
	CON (*n* = 18) -hrMCI (*n* = 8) -lrMCI (*n* = 10)	112.5 (50.2) 117.7 (58.3) 108.3 (45.5)	136.3 (66.4) 141.3 (58.3) 132.4 (75.1)	+ 21.2 + 20.1 + 22.2
	Total (*n* = 42) -hrMCI (*n* = 20) -lrMCI (*n* = 22)	116.2 (54.0) 117.9 (54.9) 114.7 (54.4)	138.0 (77.0) 142.4 (71.2) 133.9 (83.3)	+ 18.8 + 20.8 + 16.7
IL-6 (pg/mL)	EXP (*n* = 23) -hrMCI (*n* = 12) -lrMCI (*n* = 11)	8.5 (9.2) 6.7 (5.5) 10.5 (12.0)	12.2 (12.2) 10.3 (11.2) 14.4 (13.5)	+ 43.5 + 53.7 + 37.1
	CON (*n* = 20) -hrMCI (*n* = 10) -lrMCI (*n* = 10)	7.7 (8.2) 4.5 (4.2) 11.2 (10.2)	7.2 (8.9) 4.1 (3.1) 10.7 (11.8)	-6.5 -8.9 -4.5
	Total (*n* = 43) -hrMCI (*n* = 22) -lrMCI (*n *= 21)	8.1 (8.6) 5.6 (4.9) 10.8 (10.9)	9.9 (11.0) 7.3 (8.8) 12.7 (12.6)	+ 22.2 + 30.4 + 17.6
KYN (ng/mL)	EXP (*n* = 23) -hrMCI (*n* = 11) -lrMCI (*n* = 12)	1582.3 (755.7) 1434.8 (650.6) 1717.4 (846.1)	1301.3 (606.4) 1442.3 (579.5) 1172.2 (626.2)	-17.8 + 0.5 -46.5
	CON (*n* = 22) -hrMCI (*n* = 12) -lrMCI (*n* = 10)	1616.4 (709.1) 1308.2 (441.0) 2017.2 (808.8)	1507.1 (578.2) 1722.2 (593.8) 1227.5 (439.8)	-6.8 + 31.6 -39.1
	Total (*n* = 45) -hrMCI (*n* = 23) -lrMCI (*n* = 22)	1599.3 (724.8) 1366.2 (538.2) 1853.7 (823.8)	1404.2 (595.0) 1593.9 (591.8) 1197.3 (537.7)	-12.2 + 16.7 -35.4

Only the values of the participants with measurements at baseline and after 12 weeks (number of participants, *n*) were used for analysis

Abbreviations: *CON*, control; *EXP*, experimental; *hrMCI*, high risk for mild cognitive impairment; *IGF-1*, insulin-like growth factor-1; *IL-6*, interleukin-6; *KYN*, kynurenine; *lrMCI*, low risk for mild cognitive impairment

**Table 2:** Descriptive values of baseline/12 weeks hippocampus (subfield) volumes and percentage change

		BASELINE	12 WEEKS	Δ (%)
Whole hippocampus	EXP (n = 22) -hrMCI (n = 11) -lrMCI (n = 11)	3286.0 (329.0) 3181.4 (270.7) 3390.6 (360.5)	3281.6 (344.8) 3145.3 (296.5) 3417.9 (347.6)	-0.1% -1.1% + 0.8%
	CON (n = 18) -hrMCI (n = 11) -lrMCI (n = 7)	3288.3 (400.3) 3250.2 (497.9) 3348.2 (183.9)	3262.6 (395.2) 3216.8 (475.9) 3334.4 (234.8)	-0.8% -1.0% -0.4%
	Total (n = 40) -hrMCI (n = 22) -lrMCI (n = 18)	3287.0 (358.0) 3215.8 (392.7) 3374.1 (298.1)	3273.0 (363.6) 3181.1 (388.7) 3385.5 (303.8)	-0.4% -1.1% + 0.3
CA1	EXP (n = 22) -hrMCI (n = 11) -lrMCI (n = 11)	623.2 (79.3) 593.7 (54.6) 652.7 (91.2)	620.3 (83.5) 584.8 (60.6) 655.8 (90.6)	-0.5% -1.5% + 0.5%
	CON (n = 18) -hrMCI (n = 11) -lrMCI (n = 7)	620.5 (92.8) 623.6 (116.1) 615.6 (43.3)	615.6 (92.5) 618.5 (113.3) 611.0 (53.2)	-0.8% -0.8% -0.7%
	Total (n = 40) -hrMCI (n = 22) -lrMCI (n = 18)	622.0 (84.5) 608.7 (89.8) 638.3 (76.8)	618.2 (86.6) 601.7 (90.3) 638.4 (79.6)	-0.6% -1.1% + 0.0%
Subiculum	EXP (n = 22) -hrMCI (n = 11) -lrMCI (n = 11)	428.2 (49.7) 416.7 (39.2) 439.7 (58.0)	424.3 (54.3) 407.7 (44.8) 440.9 (59.8)	-0.9% -2.2% + 0.2%
	CON (n = 18) -hrMCI (n = 11) -lrMCI (n = 7)	412.9 (60.0) 399.8 (63.8) 433.4 (51.4)	413.5 (57.1) 398.9 (58.5) 436.4 (50.2)	+ 0.1% -0.2% + 0.7%
	Total (n = 40) -hrMCI (n = 22) -lrMCI (n = 18)	421.3 (54.4) 408.3 (52.4) 437.2 (54.0)	419.4 (55.1) 403.3 (51.1) 439.1 (54.8)	-0.5% -1.2% + 0.4%
Presubiculum	EXP (n = 22) -hrMCI (n = 11) -lrMCI (n = 11)	289.8 (34.0) 278.0 (35.0) 301.7 (29.9)	290.5 (38.6) 276.7 (43.4) 304.2 (28.6)	+ 0.2% -0.5% + 0.8%
	CON (n = 18) -hrMCI (n = 11) -lrMCI (n = 7)	305.0 (45.3) 294.7 (47.0) 321.2 (40.5)	304.4 (51.4) 292.2 (50.4) 323.5 (50.5)	-0.2% -0.8% + 0.7%
	Total (n = 40) -hrMCI (n = 22) -lrMCI (n = 18)	296.6 (39.7) 286.3 (41.3) 309.3 (34.7)	296.7 (44.7) 284.5 (46.6) 311.7 (38.4)	+ 0.0% -0.6% + 0.8%
CA4	EXP (n = 22) -hrMCI (n = 11) -lrMCI (n = 11)	231.0 (26.6) 222.6 (21.9) 239.4 (29.1)	231.8 (26.5) 222.0 (22.0) 241.5 (28.0)	+ 0.3% -0.3% + 0.9%
	CON (n = 18) -hrMCI (n = 11) -lrMCI (n = 7)	230.1 (29.9) 229.5 (36.7) 230.9 (17.0)	227.8 (29.5) 226.5 (35.4) 229.7 (19.0)	-1.0% -1.3% -0.5%
	Total (n = 40) -hrMCI (n = 22) -lrMCI (n = 18)	230.6 (27.7) 226.1 (29.7) 236.1 (24.9)	230.0 (27.6) 224.3 (28.9) 236.9 (25.0)	-0.3% -0.8% + 0.3%
DG	EXP (n = 22) -hrMCI (n = 11) -lrMCI (n = 11)	262.2 (32.3) 252.7 (26.3) 271.8 (36.0)	263.0 (31.8) 251.6 (26.1) 274.4 (33.9)	+ 0.3% -0.4% + 1.0%
	CON (n = 18) -hrMCI (n = 11) -lrMCI (n = 7)	263.1 (33.6) 260.6 (41.4) 267.0 (17.6)	260.0 (32.7) 257.4 (39.4) 264.1 (20.3)	-1.2% -1.2% -1.1%
	Total (n = 40) -hrMCI (n = 22) -lrMCI (n = 18)	262.6 (32.4) 256.7 (34.1) 269.9 (29.6)	261.7 (31.8) 254.5 (32.7) 270.4 (29.1)	-0.3% -0.9% + 0.2%

Only the values of the participants with measurements at baseline and after 12 weeks (number of participants, n) were used for analysis

Abbreviations: *CA*, cornu ammonis; *CON*, control; *DG*, dentate gyrus; *EXP*, experimental; *hrMCI*, high risk for mild cognitive impairment; *lrMCI*, low risk for mild cognitive impairment

**Table 3:** Descriptive values of baseline/12 weeks left hippocampus neurometabolite ratios and percentage change

		BASELINE	12 WEEKS	Δ (%)
tNAA/tCr	EXP (n = 22) -hrMCI (n = 11) -lrMCI (n = 11)	1.18 (0.16) 1.14 (0.11) 1.18 (0.19)	1.25 (0.13) 1.15 (0.18) 1.25 (0.13)	+ 5.9% + 0.9% + 5.9%
	CON (n = 13) -hrMCI (n = 5) -lrMCI (n = 8)	1.14 (0.11) 1.15 (0.11) 1.13 (0.11)	1.15 (0.18) 1.12 (0.16) 1.16 (0.20)	+ 0.9% -2.6% + 2.7%
	Total (n = 35) -hrMCI (n = 16) -lrMCI (n = 19)	1.17 (0.14) 1.17 (0.12) 1.16 (0.16)	1.21 (0.15) 1.20 (0.15) 1.22 (0.16)	+ 3.4% + 2.6% + 5.2%
mIns/tCr	EXP (n = 22) -hrMCI (n = 11) -lrMCI (n = 11)	1.06 (0.18) 1.07 (0.11) 1.03 (0.14)	1.06 (0.15) 1.03 (0.23) 1.04 (0.15)	-0.0% -3.7% + 1.0%
	CON (n = 13) -hrMCI (n = 5) -lrMCI (n = 8)	1.07 (0.11) 1.03 (0.11) 1.10 (0.10)	1.03 (0.23) 0.97 (0.25) 1.07 (0.23)	-3.7% -5.8% -2.7%
	Total (n = 35) -hrMCI (n = 16) -lrMCI (n = 19)	1.07 (0.15) 1.07 (0.18) 1.06 (0.13)	1.05 (0.18) 1.04 (0.18) 1.05 (0.18)	-1.9% -2.8% -0.9%
tNAA/mIns	EXP (n = 22) -hrMCI (n = 11) -lrMCI (n = 11)	1.13 (0.15) 1.07 (0.16) 1.15 (0.13)	1.20 (0.21) 1.20 (0.43) 1.23 (0.24)	+ 6.2% + 12.1% + 7.0%
	CON (n = 13) -hrMCI (n = 5) -lrMCI (n = 8)	1.07 (0.16) 1.12 (0.18) 1.03 (0.14)	1.20 (0.43) 1.26 (0.53) 1.15 (0.37)	+ 12.1% + 12.5% + 11.7%
	Total (n = 35) -hrMCI (n = 16) -lrMCI (n = 19)	1.11 (0.15) 1.11 (0.16) 1.10 (0.14)	1.20 (0.31) 1.20 (0.32) 1.20 (0.30)	+ 8.1% + 8.1% + 9.1%

Only the values of the participants with measurements at baseline and after 12 weeks (number of participants, n) were used for analysis

Abbreviations: *CON*, control; *EXP*, experimental; *hrMCI*, high risk for mild cognitive impairment; *lrMCI*, low risk for mild cognitive impairment; *mIns*, myo-inositol; *tCr*, total creatine; *tNAA*, total N-acetylaspartate

### Exercise-induced blood biomarker changes in cases with low versus high MCI risk

ANCOVA results did not indicate a significant group effect on the levels of IGF-1, IL-6, or KYN. However, the effect size for the IL-6 group effect was of moderate magnitude (_p_η^2^ = 0.078, p = 0.089). IL-6 levels in the exercise group increased by 43.5%, while they decreased by 6.5% in the control group. This change was most distinct in the older adults with high MCI risk, who showed IL-6 changes of + 53.7% and -8.9% in the exercise versus control group, respectively. Cognitive status significantly influenced KYN level (p = 0.015), with higher KYN levels found in high MCI risk individuals. There was no significant group x cognitive status interaction effect on IGF-1, IL-6, KYN levels. Table 1 presents the descriptive values, and Table 4 contains the two-way ANCOVA results for the blood biomarkers.

**Table 4:** ANCOVA results

	Group		Cognitive status		Group*Cognitive status	
	p-value	_p_η^2^	p-value	_p_η^2^	p-value	_p_η^2^
IGF-1	0.439	0.016	0.612	0.007	0.717	0.004
IL-6	0.089	0.078	0.401	0.020	0.476	0.014
KYN	0.372	0.021	0.015*	0.147	0.328	0.025
Whole hippocampus	0.722	0.004	0.074	0.093	0.343	0.027
CA1	0.977	0.000	0.218	0.046	0.259	0.038
Subiculum	0.190	0.052	0.043*	0.119	0.471	0.016
Presubiculum	0.728	0.004	0.483	0.015	0.991	0.000
CA4	0.171	0.056	0.227	0.044	0.677	0.005
DG	0.119	0.072	0.252	0.040	0.320	0.030
tNAA/tCr	0.072	0.107	0.835	0.002	0.808	0.002
mIns/tCr	0.942	0.000	0.538	0.013	0.894	0.001
tNAA/mIns	0.537	0.013	0.785	0.003	0.859	0.001

The dependent variables are presented in the first row. P-values and effect sizes (partial eta squared) are given for the group effect (exercise versus control), the cognitive status effect (high versus low MCI risk) and their interaction (Group*Cognitive status). Significant p-values are marked *. The ANCOVA was adjusted for the dependent variable’s baseline value and for age. The ANCOVA for IL-6 and KYN were additionally adjusted for fat%. Of note, pre-intervention KYN, post-intervention IGF-1 and IL-6, and pre- and post-intervention tNAA/mIns left hippocampal cortex were log-transformed due to a non-normal distribution, and in pre-intervention IL-6 and post-intervention IL-6 and KYN an extreme outlier was removed before analysis

Abbreviations: *CA*, cornu ammonis; *DG*, dentate gyrus; *IGF-1*, insulin-like growth factor-1; *IL-6*, interleukin-6; *KYN*, kynurenine; *mIns*, myo-inositol; *tCr*, total creatine; *tNAA*, total N-acetylaspartate; _p_η^2^ = partial eta squared

### Exercise-induced hippocampus total and subfield volume changes in cases with low versus high MCI risk

Hippocampus (subfield) volumes did not significantly differ between exercise and control group. However, the effect size for the DG group effect was of moderate magnitude (_p_η^2^ = 0.072, p = 0.119). DG volume in the exercise group increased by 0.3%, while it decreased by 1.2% in the control group. Overall, hippocampus (subfield) volume decreased over time, except for subiculum volume in the control group and the volumes of the presubiculum, CA4, and DG in the exercise group. Cognitive status had a significant influence on subiculum volume (_p_η^2^ = 0.119, p = 0.043) and an effect size of moderate magnitude was found for the total hippocampus (_p_η^2^ = 0.093, p = 0.074). In both cases, individuals with high MCI risk had lower (subfield) volumes than individuals with low MCI risk. No group x cognitive status interaction effect was observed for hippocampal volume. Table 2 contains the descriptive values and Table 4 presents the two-way ANCOVA results for (subfield) hippocampus volume.

### Exercise-induced hippocampal neurometabolite changes in cases with low and high MCI risk

There was no group or cognitive status effect nor a group x cognitive status effect for hippocampal neurometabolite changes. The effect size for the tNAA/tCr ratio group effect was of moderate magnitude (_p_η^2^ = 0.107, p = 0.072), with tNAA/tCr increasing more in the resistance exercise group than controls. Table 3 contains the descriptive values and Table 4 presents the two-way ANCOVA results for the hippocampal neurometabolite ratios.

### Correlations between blood and hippocampal changes

**Fig. 2:**
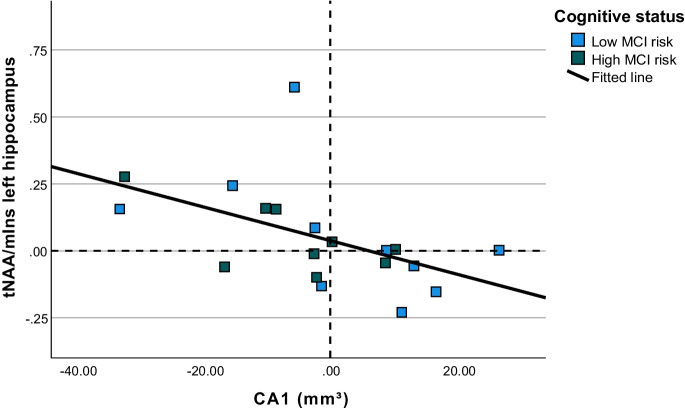
Bivariate relationship between pre-to-post changes in tNAA/mIns levels in the left hippocampus (ΔtNAA/mIns left HPC) and pre-to-post changes in CA1 volume (ΔCA1) for exercise group participants

Correlations between changes in blood biomarkers, neurometabolites, and hippocampal volume in the exercise group are reported in Table 5. Control group and total group correlations are presented in Supplementary Table 3 and 4, respectively. In the exercise group participants, CA1 volume changes showed a negative correlation with changes in hippocampal tNAA/mIns ratio (r = -0.605, p = 0.006; see also Appendix A, Fig. 2).

Positive values mark increases from pre- to post-intervention measurements. Abbreviations: CA1, cornu ammonis subfield 1; MCI, mild cognitive impairment; mIns, myoinositol; tNAA, total N-acetylaspartate.

**Table 5:** Bivariate correlations between changes in blood biomarkers, hippocampus volume and neurometabolites for exercise group participants

		ΔIL-6	ΔKYN	ΔWhole hippocampus	ΔCA1	ΔSubiculum	ΔPresubiculum	ΔCA4	ΔDG	ΔtNAA/tCr left hippocampus	ΔmIns/tCr left hippocampus	ΔtNAA/mIns left hippocampus
ΔIGF-1	R	-0.271	0.022	-0.123	-0.131	-0.128	-0.108	0.035	0.021	-0.192	-0.150	0.051
	p	0.210	0.922	0.605	0.582	0.591	0.650	0.885	0.930	0.416	0.527	0.830
ΔIL-6	R		0.155	-0.142	0.037	0.005	0.009	-0.402	-0.118	0.063	0.098	-0.144
	p		0.492	0.561	0.881	0.983	0.972	0.088	0.631	0.797	0.689	0.556
ΔKYN	R			-0.393	-0.240	-0.298	-0.030	-0.181	-0.198	0.214	-0.288	0.391
	p			0.096	0.322	0.215	0.904	0.459	0.416	0.379	0.232	0.098
ΔWhole hippocampus	R				**0.666**	**0.633**	**0.488**	**0.566**	**0.517**	-0.075	0.351	-0.401
	p				**0.001**	**0.002**	**0.021**	**0.006**	**0.014**	0.759	0.141	0.084
ΔCA1	R					**0.450**	0.404	0.120	**0.539**	-0.267	0.337	**-0.605**
	p					**0.036**	0.062	0.594	**0.010**	0.270	0.158	**0.006**
ΔSubiculum	R						0.158	0.081	0.074	0.072	0.447	-0.395
	p						0.484	0.721	0.744	0.770	0.055	0.094
ΔPresubiculum	R							0.012	-0.001	-0.044	0.151	-0.193
	p							0.958	0.998	0.858	0.538	0.429
ΔCA4	R								0.412	-0.140	-0.089	0.077
	p								0.057	0.567	0.716	0.753
ΔDG	R									-0.044	0.196	-0.284
	p									0.858	0.420	0.238
ΔtNAA/tCr left hippocampus	R										**0.558**	0.278
	p										**0.007**	0.210
ΔmIns/tCr left hippocampus	R											**-0.567**
	p											**0.006**

Significant correlations are marked in bold (*p* < 0.05). Correlations with a moderate effect size (r > 0.3) are underlined. Δ values were calculated by substracting the post-intervention value from the pre-intervention value. Spearman’s rho correlation values are presented

Abbreviations: *CA*, cornu ammonis; *DG*, dentate gyrus; *HPC*, hippocampal cortex; *IGF-1*, insulin-like growth factor-1; *IL-6*, interleukin-6; *KYN*, kynurenine; *mIns*, myo-inositol; *tCr*, total creatine; *tNAA*, total N-acetylaspartate

## Discussion

Although there is rising interest in the effects of exercise-induced peripheral biomarkers on the brain, most studies have focused primarily on aerobic exercise investigations while the number of resistance exercise studies in this field is limited [16]. Another limitation of the literature regarding the resistance exercise effect on the brain is that the majority of mechanistic explanations are derived from animal experiments. In this randomized controlled trial, we examined the effect of 12 weeks of resistance exercise on blood biomarkers, hippocampus subfield volumes, and hippocampal neurometabolites in older adults with low or high risk of MCI. The effect of resistance exercise was contrasted to a waiting list control condition. To the best of our knowledge, only one study has previously reported resistance exercise-induced neurometabolic changes. In this study from our own research group, it was discovered that strength gains following resistance exercise corresponded with neurometabolic changes in the same cohort of older adults as the current study [41]. So far, no studies have tested the relationship between these exercise-responsive blood and brain neurochemical biomarkers, while this could provide important insights into the mechanism behind the exercise-cognition relationship.

As our primary outcome, we discovered that pre-to-post exercise changes in volume of the CA1 were negatively associated with pre-to-post exercise changes in ratios of tNAA/mIns in the hippocampus. This indicates that the more the density of neurons (marked by tNAA) relative to the density of glial cells (marked by mIns) increases following exercise, the larger the CA1 volume loss following exercise. An interpretation could be that either exercise increases the density of hippocampal neurons at the expense of CA1 volume or an exercise-induced increase in glial cells is associated with CA1 volume gain. The former interpretation is not in line with previous studies showing that exercise can attenuate (subfield) hippocampal volume loss (e.g. [58–60]). However, from the neurometabolic perspective, it does fit our finding that hippocampal tNAA/tCr levels tended to increase in the resistance exercise group. The latter interpretation also does not fit our initial hypothesis that mIns would be associated with neuroinflammation, which at high levels is detrimental for neuroplasticity [14, 61]. The relationship between inflammatory cells and neuroplasticity is however much more complex than initial studies had pointed out [62]. Research has shown that at least some inflammation is in fact essential for neuroplastic processes [14], and microglial cells can support neuroplasticity [62]. In our study, there was a general decrease in hippocampal mIns/tCr levels in the control group, while hippocampal mIns/tCr levels remained the same in the exercise group. This difference was not significant. Trends from our correlation analysis with moderate effect size (r > 0.3) support a possible role of mIns (glial cells) in hippocampus volume increases. Specifically, we found a triad relationship between lower KYN levels, lower tNAA/mIns or higher mIns/tCr levels, and total hippocampal volume. Additionally, we found a relationship between lower IL-6 levels and higher CA4 volume and higher mIns/tCr levels and higher CA4 volume. The inverse relationships between our inflammatory blood biomarkers and hippocampus (subfield) volumes is in line with our initial hypothesis. Our results suggest that mIns level changes (glial cells) may also play a role in the inverse relationship between blood inflammatory markers and hippocampal (subfield) volumes. To the best of our knowledge, there is no previous evidence of exercise-induced changes in mIns levels. Again, in our cohort of older adults, no significant group effect was found for mIns/tCr levels in the hippocampus, nor for the left sensorimotor cortex or right dorsolateral prefrontal cortex, as was previously reported by Sheoran et al. [41]. Furthermore, we did not find an effect of resistance exercise on tNAA/tCr or tNAA/mIns levels in the hippocampus, left sensorimotor cortex or right dorsolateral prefrontal cortex [41]. However, hippocampal tNAA/tCr level group differences had a moderate effect size, suggesting that the resistance exercise group tended to increase tNAA/tCr in the hippocampus. Similar to our results, a study with a 12-week aerobic exercise program also reported no significant exercise-induced changes in tNAA in older adults [63]. However, tNAA increases were reported in other aerobic exercise studies with the same exercise duration in middle-aged adults [64], Den [65]. Notably, only few studies have examined neurometabolic changes caused by physical exercise and it remains unexplored if exercise-induced changes in brain neurometabolic properties precede, follow or coincide with volumetric changes. Studies with longer resistance exercise programs and repeated measurements are needed to discover if tNAA increases may follow at a later stage of physical exercise-induced neuroplastic changes in older adults, and if mIns may indeed play a role in the initial stages of exercise-induced hippocampal neurochemical and volumetric changes or if mIns levels may decrease at a later stage as we hypothesized.

Secondarily, we investigated resistance exercise-induced effects on blood and hippocampus biomarkers. ANCOVA test on the 12-week differences between groups, while taking into account the baseline values, did not indicate any significant effects. However, a nonsignificant difference with a moderate effect size was found for IL-6, with higher levels in the resistance exercise group compared to the control group, especially in older adults with high MCI risk. These findings were in contrast to our hypothesis, as a previous meta-analysis has reported that exercise, including also resistance exercise, can induce decreases in inflammatory markers such as IL-6 [66]. Notably, KYN levels did not show the same trend. A possible interpretation for the increase in IL-6 could be that our resistance exercise program was too intense for the participating older adults (with high MCI risk). The load lifted during our training protocol was constantly adjusted to stay between 7 and 10/10 RPE, indicating that the training stimulus subjectively felt as a very difficult to maximal effort for the older adults [54]. Indeed, overtraining is known to be associated with elevated inflammation [67]. Furthermore, the time-related decreases of IL-6 in the control group were also unexpected, as aging has been linked to increases in inflammatory marker levels [15]. However, a meta-analysis has shown that elevated inflammatory markers are not always reported in older adults with MCI, while being found consistently in persons with Alzheimer’s dementia, suggesting that systemic inflammation could be a marker for Alzheimer’s dementia diagnosis [68]. Furthermore, a nonsignificant group effect with moderate effect size was found for DG volume changes, showing a slight increase in the resistance exercise group, but a decrease in the control group. We found only one previous randomized controlled trial that examined changes in subfield hippocampus volumes following resistance exercise [58]. In this study, 18 months of resistance exercise stopped atrophy of the left subiculum, and attenuated atrophy in the left CA1 and DG compared to sham physical training in older adults with MCI [58]. A 24-month multimodal exercise intervention consisting of walking at moderate intensity, lower extremity resistance exercises, balance exercises, stretching and behavioral counselling also increased total left hippocampus volume when adjusting for baseline volumes, while also the post-intervention total right hippocampus volume and volume of the left CA region differed significantly between intervention and control group when not adjusted for baseline volume [59]. The lack of total hippocampus volume changes in our study was in line other studies. For example, 6 months of resistance exercise did not change hippocampus volumes in older women with probable MCI, while these changes were found in the aerobic exercise group in this study (Ten [69]). Moreover, a resistance exercise study with a longer duration of 12-months, comparing heavy or moderate intensity resistance exercise with a control group, reported age-related hippocampal volume losses in all groups without influence of strength training [70].

Finally, when comparing older adults with low and high MCI risk, we found a significant difference in KYN levels and subiculum post-intervention volume. KYN can be considered a generic pro-inflammatory marker, as the enzyme that converts tryptophan to kynurenine in the liver, indoleamine 2,3 dioxygenase, is upregulated by pro-inflammatory cytokines, like IL-1β, tumour necrosis factor-α (TNF-α) and interferon-γ (IFN-γ) [27]. In addition, we previously reported associations between KYN levels and neuroinflammation and neurodegeneration measured with ^1^H-MRS [21], suggesting KYN can serve as a surrogate marker of brain health. In addition to our results, higher KYN levels were associated with worse cognitive test scores in a previous study in older adults [26]. Concerning hippocampus subfield volume differences depending on cognitive status, a previous meta-analysis evaluating whole brain changes caused by MCI has shown largest GMV decreases in the hippocampus, parahippocampal gyrus and amygdala in MCI compared to healthy older adults [71]. Some authors have argued that CA1 [72, 73] or subiculum [74, 75] volumes are even better markers of MCI than whole hippocampal volume. The latter subfield was also influenced by MCI risk in our study.

A limitation of this study is that it took place during the COVID-19 pandemic. This not only caused additional drop-outs from the study, COVID-19 may cause neurological symptoms, brain structural and neurochemical changes [76, 77]. A separate pre-to-post analysis of the COVID-19 cases in our study (here these participants were excluded) is currently under review (unpublished).

## Conclusion

In conclusion, this randomized controlled trial investigated the effects of 12 weeks resistance exercise compared to a waiting list control group on changes in blood biomarkers, hippocampus subfield volumes, hippocampal neurometabolites and their interrelationship in older adults with low or high risk of MCI. The study found a negative association between resistance exercise-induced changes in CA1 volume and hippocampal tNAA/mIns levels in the exercise group, possibly indicating a role of glial cells in exercise-induced neuroplasticity. Furthermore, we found a significant difference for KYN levels and subiculum volume between older adults with low compared to high MCI risk. These findings broaden the knowledge on the multifactorial effects of resistance exercise in older adults and the influence of cognitive status. Ultimately, this study may serve future work on designing individualized exercise interventions aiming to improve brain health in older adults with or without MCI or prevent age-related cognitive decline. Further investigation is warranted to assess the potential benefits that older adults may derive from a resistance exercise program with alternative intensity levels compared to the intensity used in the present protocol. Additionally, it would be of great value to explore the necessity of a longer duration of resistance exercise programs to elicit more pronounced changes.

### Supplementary Information

Below is the link to the electronic supplementary material.

Supplementary file1 (DOCX 255 KB)

Supplementary file2 (DOCX 22 KB)

## Data Availability

The coded data can be made available upon reasonable request and after approval by the Kaunas Regional Biomedical Research Ethics Committee.

## Abbreviations

^*1*^*H-MRS*: Proton magnetic resonance spectroscopy
*1 RM*: One repetition maximum
*BMI*: Body mass index
*CA*: Cornu ammonis
*DG*: Dentate gyrus
*ELISA*: Enzyme-linked immunosorbent assay
*fat%*: Body fat percentage
*GMV*: Grey matter volume
*HPC*: Hippocampal cortex
*IFN-γ*: Interferon-γ
*IGF-1*: Insulin-like growth factor-1
*IL-6*: Interleukin-6
*IPAQ-SF*: International Physical Activity Questionnaire-Short Form
*KYN*: Kynurenine
*LTP*: Long-term synaptic potentiation
*MCI*: Mild cognitive impairment
*mIns*: Myo-inositol
*MoCA*: Montreal Cognitive Assessment
*tCr*: Total creatine
*tNAA*: Total N-acetylaspartate
*TNF-α*: Tumor necrosis factor-α
