# Supplementary material for: Resistance exercise effects on hippocampus subfield volumes and biomarkers of neuroplasticity and neuroinflammation in older adults with low and high risk of mild cognitive impairment: a randomized controlled trial
Source: GeroScience. 2024 Mar 13;46(4):3971–91. doi: 10.1007/s11357-024-01110-6 (PMC11226571; doi:10.1007/s11357-024-01110-6)
Supplement: Supplementary file 1 — Supplementary file1 (DOCX 255 KB) [file 11357_2024_1110_MOESM1_ESM.docx]

**Appendix A. Supplementary Figures and Tables**


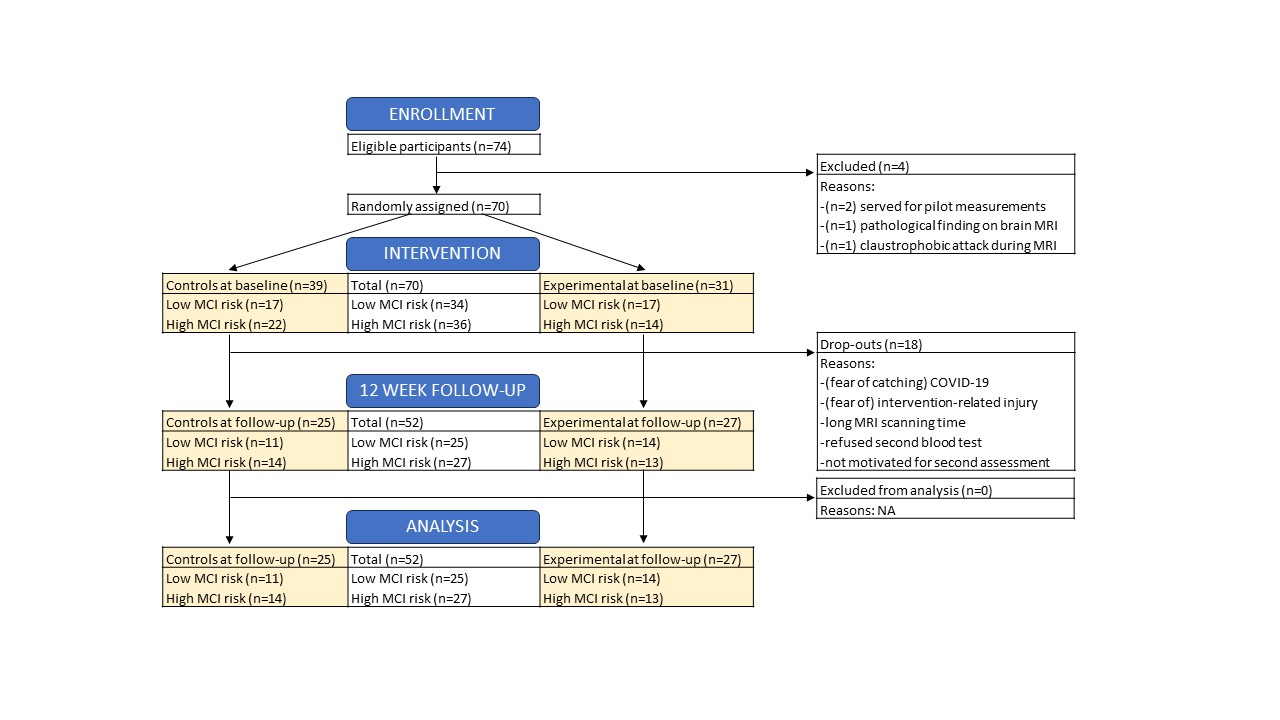


**Supplementary Figure 1.** Participant flow diagram

Abbreviations: MCI, mild cognitive impairment; MRI, magnetic resonance imaging; NA, not applicable.


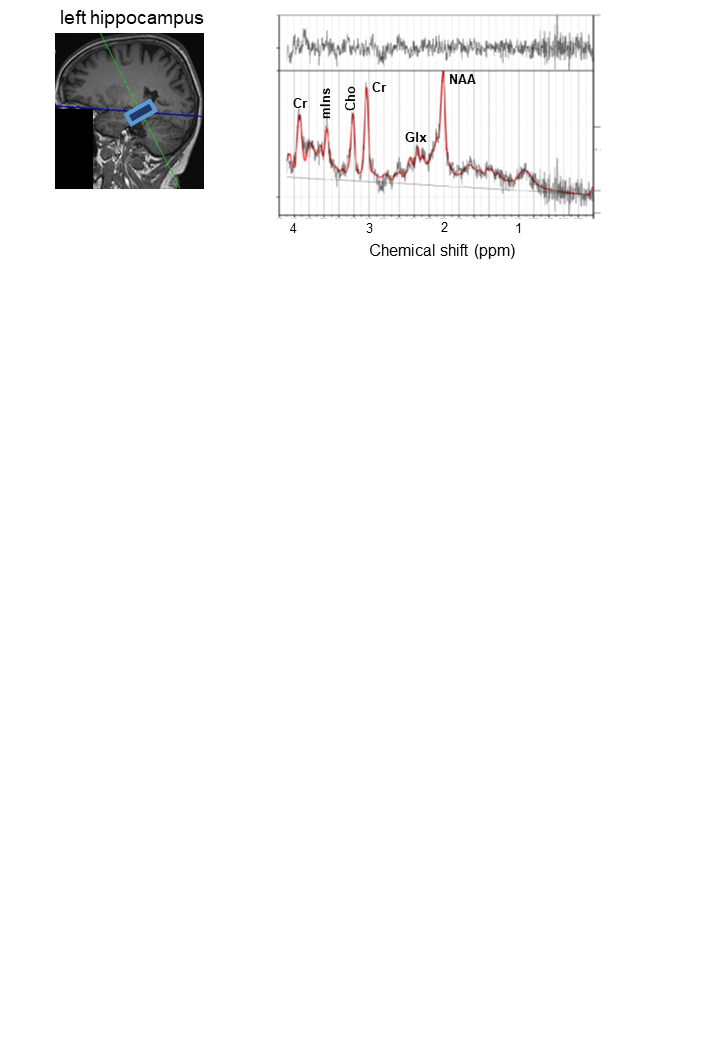


**Supplementary Figure 2.** ^1^H-MRS voxel placement and example of a post-processed spectrum

| **Supplementary Table 1**. Baseline participant characteristics and group differences | | | | | |
| --- | --- | --- | --- | --- | --- |
|  | Missing | Control  (n = 25) | Experimental  (n = 27) | Total  (n=52) | p-value |
| Age | 0 | 69.0 (5.9) | 70.7 (5.6) | 69.9 (5.8) | 0.293 |
| Sex:  - Male  - Female | 0 | 12 (48.0%)  13 (52.0%) | 12 (44.4%)  15 (55.6%) | 24 (46.2%)  28 (53.8%) | 0.797 |
| Education:  - Higher  - Secondary  - Basic | 0 | 22 (88.0%)  1 (4.0%)  2 (8.0%) | 20 (74.1%)  7 (25.9%)  0 (0%) | 42 (80.8%)  8 (15.4%)  2 (3.8%) | 0.038* |
| Smoking status  - Smoker | 0 | 1 (4.0%) | 2 (7.4%) | 3 (5.8%) | 1.000 |
| IPAQ-SF kcal/week | 0 | 5759.8 (4542.3) | 3296.7 (2711.3) | 4480.9 (3873.1) | 0.024* |
| IPAQ-SF PA level:  - sedentary  - moderately active  - highly active | 0 | 1 (4.0%)  8 (32.0%)  16 (64.0%) | 4 (14.8%)  13 (48.1%)  10 (37.0%) | 5 (9.6%)  21 (40.4%)  26 (50.0%) | 0.116 |
| BMI (kg/m²) | 1 | 27.4 (3.3) | 28.4 (4.6) | 28.1 (5.0) | 0.385 |
| Fat % | 1 | 31.1 (8.0) | 32.2 (9.6) | 31.5 (9.4) | 0.656 |
| Right hand grip strength (kg) | 1 | 34.1 (11.6) | 32.3 (8.6) | 33.2 (10.1) | 0.536 |
| MVC (Nm) | 21 | 158.1 (32.4) | 141.6 (38.5) | 149.0 (36.3) | 0.212 |
| MoCA score | 0 | 24.5 (3.4) | 25.6 (2.5) | 25.0 (3.0) | 0.204 |
| High MCI risk |  | 14 (56.0%) | 13 (48.1%) | 27 (51.9%) | 0.571 |
| Continuous parameters are expressed as mean values (SD), p-values are derived from independent t-tests; categorical parameters are expressed as n (% of total), p-values are derived from Chi² tests or Fisher Exact tests. Significant p-values are marked *.  Abbreviations: BMI, body mass index; IPAQ-SF, International Physical Activity Questionnaire-Short Form; MCI, mild cognitive impairment; MoCA, Montreal Cognitive Assessment | | | | | |

| **Supplementary Table 2.** Baseline participant characteristics and differences between older adults with low and high MCI risk | | | | |
| --- | --- | --- | --- | --- |
|  | Low MCI risk  (n = 25) | High MCI risk  (n = 27) | Total  (n=52) | p-value |
| Age | 69.0 (5.7) | 70.7 (5.9) | 69.9 (5.8) | 0.293 |
| Sex:  - Male  - Female | 10 (40.0%)  15 (60.0%) | 14 (51.9%)  13 (48.1%) | 24 (46.2%)  28 (53.8%) | 0.392 |
| Education:  - Higher  - Secondary  - Basic | 21 (84.0%)  4 (16.0%)  0 (0.0%) | 21 (77.8%)  4 (14.8%)  2 (7.4%) | 42 (80.8%)  8 (15.4%)  2 (3.8%) | 0.382 |
| Smoking status  - Smoker | 2 (8.0%) | 1 (3.7%) | 3 (5.8%) | 0.603 |
| IPAQ-SF kcal/week | 4108.9 (3208.8) | 4825.3 (4434.5) | 4480.9 (3873.1) | 0.511 |
| IPAQ-SF PA level:  - sedentary  - moderately active  - highly active | 1 (4.0%)  11 (44.0%)  13 (52.0%) | 4 (14.8%)  10 (37.0%)  13 (48.1%) | 5 (9.6%)  21 (40.4%)  26 (50.0%) | 0.412 |
| BMI (kg/m²) | 28.1 (4.1) | 27.7 (3.9) | 28.1 (5.0) | 0.679 |
| Fat % | 33.2 (7.5) | 30.3 (9.8) | 31.5 (9.4) | 0.242 |
| Right hand grip strength (kg) | 32.9 (9.1) | 33.4 (11.1) | 33.2 (10.1) | 0.882 |
| MVC (Nm) | 150.6 (35.0) | 145.2 (41.1) | 149.0 (36.3) | 0.712 |
| IGF-1 | 114.1 (52.2) | 132.9 (58.5) | 116.2 (54.0) | 0.234 |
| IL-6 | 9.6 (10.7) | 5.8 (4.8) | 8.1 (8.6) | 0.117 |
| KYN | 1767.0 (848.0) | 1488.3 (742.2) | 1599.3 (724.8) | 0.217 |
| Whole hippocampus | 3374.1 (298.1) | 3215.8 (392.7) | 3287.0 (358.0) | 0.167 |
| CA1 | 638.3 (76.8) | 608.7 (89.8) | 622.0 (84.5) | 0.275 |
| Subiculum | 437.2 (54.0) | 408.3 (52.4) | 421.3 (54.4) | 0.095 |
| Presubiculum | 309.3 (34.7) | 286.3 (41.3) | 296.6 (39.7) | 0.068 |
| CA4 | 236.1 (24.9) | 226.1 (29.7) | 230.6 (27.7) | 0.259 |
| DG | 269.9 (29.6) | 256.7 (34.1) | 262.6 (32.4) | 0.203 |
| tNAA/tCr | 1.17 (0.15) | 1.17 (0.11) | 1.17 (0.14) | 0.869 |
| mIns/tCr | 1.09 (0.14) | 1.05 (0.23) | 1.07 (0.15) | 0.406 |
| tNAA/mIns | 1.08 (0.14) | 1.16 (0.23) | 1.11 (0.15) | 0.198 |

| **Supplementary Table 3.** Bivariate correlations between changes in blood biomarkers, hippocampus volume and neurometabolites for control group participants | | | | | | | | | | | |
| --- | --- | --- | --- | --- | --- | --- | --- | --- | --- | --- | --- |
|  | ΔIL-6 | ΔKYN | ΔWhole hippocampus | ΔCA1 | ΔSubiculum | ΔPresubiculum | ΔCA4 | ΔDG | ΔtNAA/tCr left HPC | ΔmIns/tCr left HIPC | ΔtNAA/mIns left HPC |
| ΔIGF-1 | 0.018 | -0.015 | -0.346 | 0.165 | **-0.681** | **-0.593** | -0.022 | 0.214 | 0.098 | -0.455 | 0.364 |
| ΔIL-6 |  | -0.300 | 0.421 | 0.341 | 0.082 | **0.559** | 0.159 | 0.062 | 0.191 | 0.200 | -0.045 |
| ΔKYN |  |  | -0.051 | 0.073 | 0.030 | -0.092 | 0.022 | 0.201 | -0.343 | 0.210 | -0.357 |
| ΔWhole hippocampus |  |  |  | **0.761** | **0.740** | **0.759** | **0.736** | 0.455 | -0.139 | 0.261 | -0.176 |
| ΔCA1 |  |  |  |  | 0.389 | 0.366 | **0.686** | **0.695** | -0.406 | 0.467 | -0.455 |
| ΔSubiculum |  |  |  |  |  | **0.645** | 0.366 | 0.160 | -0.079 | 0.091 | -0.103 |
| ΔPresubiculum |  |  |  |  |  |  | 0.269 | -0.073 | 0.212 | 0.236 | -0.079 |
| ΔCA4 |  |  |  |  |  |  |  | **0.602** | -0.576 | 0.103 | -0.200 |
| ΔDG |  |  |  |  |  |  |  |  | -0.345 | 0.103 | -0.079 |
| ΔtNAA/tCr left HPC |  |  |  |  |  |  |  |  |  | -0.253 | **0.588** |
| ΔmIns/tCr left HPC |  |  |  |  |  |  |  |  |  |  | **-0.874** |
| Significant correlations are marked in bold (p < 0.05). Δ values were calculated by substracting the post-intervention value from the pre-intervention value. Spearman’s rho correlation values are presented.  Abbreviations: CA, cornu ammonis; DG, dentate gyrus; HPC, hippocampal cortex; IGF-1, insulin-like growth factor-1; IL-6, interleukin-6; KYN, kynurenine; mIns, myo-inositol; tCr, total creatine; tNAA, total N-acetylaspartate. | | | | | | | | | | | |

| **Supplementary Table 4.** Bivariate correlations between changes in blood biomarkers, hippocampus volume and neurometabolites for all participants | | | | | | | | | | | |
| --- | --- | --- | --- | --- | --- | --- | --- | --- | --- | --- | --- |
|  | ΔIL-6 | ΔKYN | ΔWhole hippocampus | ΔCA1 | ΔSubiculum | ΔPresubiculum | ΔCA4 | ΔDG | ΔtNAA/tCr left HPC | ΔmIns/tCr left HIPC | ΔtNAA/mIns left HPC |
| ΔIGF-1 | -0.148 | -0.018 | -0.198 | 0.039 | -0.333 | -0.295 | -0.027 | 0.009 | -0.019 | -0.241 | 0.188 |
| ΔIL-6 |  | -0.063 | 0.176 | 0.165 | 0.041 | 0.314 | -0.041 | 0.093 | 0.096 | 0.133 | -0.083 |
| ΔKYN |  |  | -0.199 | -0.081 | -0.064 | -0.069 | -0.152 | -0.226 | -0.033 | -0.062 | 0.022 |
| ΔWhole hippocampus |  |  |  | **0.727** | **0.717** | **0.611** | **0.650** | **0.741** | -0.141 | 0.300 | -0.306 |
| ΔCA1 |  |  |  |  | **0.446** | **0.352** | **0.404** | **0.577** | -0.279 | **0.437** | **-0.546** |
| ΔSubiculum |  |  |  |  |  | **0.434** | 0.211 | 0.298 | -0.007 | 0.320 | -0.275 |
| ΔPresubiculum |  |  |  |  |  |  | 0.190 | 0.214 | 0.087 | 0.152 | -0.122 |
| ΔCA4 |  |  |  |  |  |  |  | **0.903** | -0.331 | 0.033 | -0.072 |
| ΔDG |  |  |  |  |  |  |  |  | -0.209 | 0.072 | -0.063 |
| ΔtNAA/tCr left HPC |  |  |  |  |  |  |  |  |  | 0.232 | **0.416** |
| ΔmIns/tCr left HPC |  |  |  |  |  |  |  |  |  |  | **-0.704** |
| Significant correlations are marked in bold (p < 0.05). Δ values were calculated by substracting the post-intervention value from the pre-intervention value. Spearman’s rho correlation values are presented.  Abbreviations: CA, cornu ammonis; DG, dentate gyrus; HPC, hippocampal cortex; IGF-1, insulin-like growth factor-1; IL-6, interleukin-6; KYN, kynurenine; mIns, myo-inositol; tCr, total creatine; tNAA, total N-acetylaspartate. | | | | | | | | | | | |
