# Supplementary material for: Resistance exercise effects on hippocampus subfield volumes and biomarkers of neuroplasticity and neuroinflammation in older adults with low and high risk of mild cognitive impairment: a randomized controlled trial
Source: GeroScience. 2024 Mar 13;46(4):3971–91. doi: 10.1007/s11357-024-01110-6 (PMC11226571; doi:10.1007/s11357-024-01110-6)
Supplement: Supplementary file 2 — Supplementary file2 (DOCX 22 KB) [file 11357_2024_1110_MOESM2_ESM.docx]

**Appendix B:** **MRSinMRS Reporting Checklist for single-voxel MR spectroscopy imaging^1^**

| 1. Hardware 2. Field strength: 3T 3. Manufacture: Siemens 4. Model: Skyra (VE11E) 5. RF coil: 32 channels ^1^H- head coil 6. Acquisition 7. Pulse sequence: PRESS 8. Volume of interest (VOI) location: 9. left hippocampus (left HPC) 10. Nominal VOI size: 11. 26 × 12 × 12 mm^3^ 12. Repetition time (T_R_) and echo time (T_E_) 13. T_R_ = 2000 ms, 14. T_E_ = 30 ms 15. Total number of acquisitions per spectrum: 128 averages 16. Spectral bandwidth: 2000 Hz 17. Number of spectral points: 1024 points 18. Water suppression method: CHESS (bandwidth 50 Hz)) 19. Shimming method: Automated B0-field mapping followed by manual adjustment to reduce water signal FHWM below 15Hz |
| --- |
| 1. Analysis software and outputs 2. Analysis software: LCModel 6.3.1-R 3. Output measures: Ratio to total creatine (tCr) of total NAA (tNAA = NAA + NAAG), total choline (tCho = GPC + PCh), myo-inositol (mIns), and glutamate-glutamine complex (Glx = Glu + Gln) and ratio to mIns of tNAA 4. Processing steps: estimation of water-referenced values of tCr, tNAA, tCho, mIns, and Glx |
| 1. Fitting model basis set   27 basis spectra including: alanine (Ala), aspartate (Asp), creatine (Cr), phosphocreatine (PCr), γ-aminobutyric acid (GABA), glucose (Glc), glutamine (Gln), glutamate (Glu), glycerophosphocholine (GPC), phosphorylcholine (PCh), myo-inositol (mIns), lactate (Lac), N-acetyl aspartate (NAA), N-acetyl-aspartyl-glutamate (NAAG), scyllo-Inositol (Scyllo), taurine (Tau), negative creatine methylene (-CrCH2), guanidinoacetate (Gua), lipids [Lip09, Lip13a, Lip13b, and Lip20] and macromolecules [MM09, MM12, MM14, MM17 and MM20] |
| 1. Data quality 2. Data exclusion criteria: SNR < 5, FWHM ≥ 15 Hz or CRLB ≥ 20 % 3. Reported measures of SNR and FWHM (in ppm) as reported by LCModel for included spectra:   i. left HPC: SNR [8.75 ± 1.69 (6-12)], FWHM [0.076 ± 0.014 (0.043 – 0.105) ppm]   1. CRLB as reported by LCModel for included spectra:   i. left HPC: tNAA [5.83 ± 1.20 (3-8) %], mIns [5.31 ± 1.19 (3-9) %], tCr [4.36 ± 0.74 (3-6) %] |
| **^1^**In line with the guidelines presented in Lin A et al. (2021) |
